# Notch-Hes1 Signaling Regulates IL-17A^+^*γδ*^+^T Cell Expression and IL-17A Secretion of Mouse Psoriasis-Like Skin Inflammation

**DOI:** 10.1155/2020/8297134

**Published:** 2020-05-12

**Authors:** Yanqin Wang, Xinxin Li, Xiaoyun Xing, Haibo Xue, Ruiqun Qi, Hong Ji, Lei Ma

**Affiliations:** ^1^Department of Dermatology, Binzhou Medical University Hospital, 661 Second Huanghe Road, Binzhou 256603, China; ^2^Department of Endocrinology and Metabolism, Binzhou Medical University Hospital, 661 Second Huanghe Road, Binzhou 256603, China; ^3^Department of Dermatology, The First Affiliated Hospital of China Medical University, 155 North Nanjing Road, Shenyang 110001, China; ^4^Department of Pathology, Binzhou Medical University Hospital, 661 Second Huanghe Road, Binzhou 256603, China

## Abstract

**Purpose:**

To evaluate the regulating effect of Notch-Hes1 signaling on IL-17A^+^*γδ*^+^T cell expression and IL-17A secretion in mouse psoriasis-like skin inflammation.

**Materials and Methods:**

Experimental mice were randomly divided into control group, model group (5% imiquimod- (IMQ-) treated mice), and intervention group (IMQ and *γ*-secretase inhibitor DAPT cotreated mice). The severity of psoriasis-like skin inflammation was evaluated by target lesion score based on the clinical psoriasis area and severity index (PASI). Flow cytometry detected IL-17A^+^*γδ*^+^T cell percentage. Quantitative real-time RT-PCR detected Hes1 mRNA expression. Enzyme-linked immunosorbent assay and western blot measured IL-17A serum concentration and protein expression. Additionally, splenic single cells from model mice were treated by DAPT to further evaluate the inhibitory effect of blocking Notch-Hes1 signaling on IL-17A^+^*γδ*^+^T cell differentiation and IL-17A secretion.

**Results:**

The spleen index, IL-17A^+^*γδ*^+^T cell percentage, Hes1 mRNA expression, IL-17A serum concentration, and protein expression were all significantly higher in model mice than control mice, while dramatically reduced in intervention mice by DAPT treatment, which also obviously alleviated the target lesion score, epidermal hyperplasia, and dermal inflammatory cell infiltration of intervention mice. In vitro study demonstrated that DAPT treatment could result in dose-dependent decrease of IL-17A^+^*γδ*^+^T cell percentage and IL-17A secretion in splenic single cells of model mice.

## 1. Introduction

Psoriasis is an immune-mediated chronic, recurrent, and inflammatory skin disorder, which is characterized by epidermal proliferation and massive infiltration of immune cells, producing lots of cytokines, chemokines, and inflammatory molecules [1]. Although the pathogenesis of psoriasis has not been fully understood, growing evidence indicated that the cytokine interleukin- (IL-) 17 plays key roles in the development of psoriasis [2–4]. And in the animal model of mouse psoriasis-like skin inflammation, the critical roles of IL-17 have also been confirmed [5]. The IL-17 cytokine family consists of six members, i.e., IL-17A to F, among which IL-17A is classically considered to be the most biologically active. Clinical trials have demonstrated that biopharmaceuticals neutralizing IL-17A were highly efficacious for moderate to severe plaque psoriasis [6–12]. Previous studies suggested that IL-17A was mainly produced by Th17 cells in psoriatic lesions [13, 14]. Besides Th17 cells, other types of IL-17A-producing cells have also been found in the skin, including CD8^+^ T cells (Tc17), innate lymphoid cells, and *γδ*T cells [15–18]. Moreover, *γδ*T cells have been recently reported to be a major IL-17A producer in human psoriatic lesion and mouse psoriasis-like skin inflammation [18].

Notch signaling is a highly conserved signal transduction pathway, which plays a critical role in cell differentiation and proliferation, and influences cell fate decision in multiple organisms and tissues, including epidermis and its appendages [19, 20]. The mammalian Notch signaling pathway consists of four Notch receptors (Notch 1, 2, 3, and 4) and five Notch ligands (Jagged-1 and Jagged-2 and Delta-like 1, 3, and 4). In psoriatic lesions, Notch signaling molecules have been found to be highly expressed with a frequent and diffuse mode [21]. In addition, Notch signaling is hyperactivated and participated in the regulation of keratinocyte proliferation and differentiation and vascular endothelial cell function as well as early T cell development in the thymus and modulation of peripheral T cell differentiation [22–27]. Hes1, an important downstream target gene of Notch signaling, has been reported to be critically involved in the development of IL-17-producing *γδ*T cells [28]. To determine whether Notch-Hes1 signaling can regulate the function of IL-17A^+^*γδ*^+^T cells within the disease situation of psoriasis, we inhibit Notch-Hes1 signaling by *γ*-secretase inhibitor DAPT to determine the possible regulating function of Notch-Hes1 signaling on IL-17A^+^*γδ*^+^T cell expression and IL-17A secretion in mouse psoriasis-like skin inflammation.

## 2. Materials and Methods

### 2.1. Experimental Mice

Male BALB/c mice aged 6 weeks and weighted 18 ± 2 g were purchased from Jinan Pengyue Laboratory Animal Breeding Co. Ltd. (Jinan China) and bred in specific pathogen-free environment in the animal center of Binzhou Medical University Hospital. Experimental mice were randomly divided into control group (*n* = 10), model group (*n* = 10), and intervention group (*n* = 10). All animal procedures were approved by the Laboratory Animal Ethics Committee of Binzhou Medical University Hospital. In the model group and intervention group, mice received a daily topical dose of 62.5 mg of commercially available 5% imiquimod (IMQ) cream (3M Health Care Limited, UK) on shaved back to induce psoriasis-like skin inflammation, and an equivalent amount of vaseline was applied on control mice. In the intervention group, DAPT dissolved in corn oil (10 mg/kg/day, Sigma-Aldrich, St Louis, MO, USA) was intraperitoneally injected since the beginning of IMQ application to block Notch-Hes1 signaling. The control group and model group were intraperitoneally injected an equivalent amount of corn oil. After consecutive 6 days, all mice were anesthetized. Blood was collected by heart puncture; at the same time, spleen and skin tissues were acquired to complete the following experiment.

### 2.2. Skin Structural Character Observation and Histopathological Examination

The changes of skin structural characters were observed daily, and the severity of psoriasis-like skin inflammation was evaluated by the target lesion score based on the clinical psoriasis area and severity index (PASI), except that the affected skin area is not taken into account in the overall score [5]. Erythema, scaling, and thickening were scored independently on a scale from 0 to 4: 0, none; 1, slight; 2, moderate; 3, marked; and 4, very marked. The cumulative score (erythema plus scaling plus thickening) served as a measure of the severity of inflammation (scale 0–12). Skin samples were fixed in 10% neutral formalin, embedded with paraffin, sectioned, and stained with haematoxylin and eosin (HE). Epidermal thickness was measured using Image-Pro Plus 6.0 imaging system. Histopathological changes were evaluated by well-trained pathologists in a double-blind fashion.

### 2.3. Preparation of Single Cell Suspension from Spleen and Skin Tissues

Spleen tissues were fragmented into small pieces and pressed against a 200-gauge steel mesh. Cell suspension was collected, and erythrocytes were lysed by red cell lysis buffer (Sangon Biotech Shanghai Co. Ltd., Shanghai, China). Cells were resuspended and adjusted to a concentration of 1 × 10^6^/ml in Dulbecco's Modified Eagle Medium (DMEM) (Sangon, China) containing 15% fetal bovine serum and 1% penicillin and streptomycin.

Skin tissues were cut into 0.5 cm × 0.5 cm pieces and soaked in 0.5% trypsin (Sigma-Aldrich, USA) at 37°C for 2 hr. After separating the dermis and epidermis, the dermis was shaken and digested with DMEM containing collagen enzyme IV (Sigma-Aldrich, USA) and deoxyribonucleic acid enzyme I (DNase I) (Thermo, USA) at 90 rpm for 1 hr. Then, cells were resuspended and adjusted to a concentration of 1 × 10^6^/ml.

### 2.4. Splenic Single Cell Treatment by DAPT

Isolated splenic single cells from model mice were divided into DMSO control group and DAPT-treated groups (each *n* = 6) at desired concentrations of 2.5, 5, 10, and 20 *μ*mol/l, respectively, and cultured for 72 hr at 37°C, 5% CO_2_ environment. Then, about 1 × 10^6^ cells were plated in 24-well flat plate and polarized under the following situation for 24 hr, consisting of 5 *μ*g/ml CD3 monoclonal antibody (mAb), 10 *μ*g/ml CD28 mAb, 50 ng/ml recombinant IL- (rIL-) 1*β*, 50 ng/ml rIL-23, 50 ng/ml rIL-6, and 1 ng/ml TGF-*β*. All the antibodies and recombinant cytokines were from eBioscience company (San Diego, CA, USA). After polarization and stimulation, cells were collected and used for flow cytometric analysis. In addition, the cell-free supernatant was harvested for IL-17A detection by enzyme-linked immunosorbent assay (ELISA).

### 2.5. Flow Cytometric Analysis of IL-17A^+^*γδ*^+^T Cell Percentage (IL-17A^+^*γδ*^+^T Cells/*γδ*^+^T Cells%)

For IL-17A^+^*γδ*^+^T cell percentage detection, 1 × 10^6^ cells/ml single cell suspension were firstly stimulated with the Cell Stimulation Cocktail (eBioscience, San Diego, CA, USA) at 37°C under a 5% CO_2_ environment for 4 hr. Next, cells were collected, washed, and surface-stained with FITC-labeled CD3 antibody and APC-labeled *γδ*T antibody (eBioscience, USA) at 4°C in the dark for 15 min. Then, cells were fixed, permeabilized, and stained intracellularly with PE-labeled IL-17A antibody (eBioscience, USA) at 4°C in the dark for 20 min. Flow cytometric analysis was performed on a FACScanto flow cytometer (BD Biosciences, San Jose, CA, USA).

### 2.6. Quantitative Real-Time RT-PCR Analysis of Hes1 mRNA Expression

RNA of splenic single cell suspension and skin tissues were extracted by Trizol reagent (Invitrogen, Carlsbad, CA, USA). RNA integrity and purity were verified by performing 1.5% agarose gel electrophoresis of intact 28S and 18S rRNA bands with a 2 : 1 ratio and spectrophotometer (A260/A280 ratio) of 1.8-2.0. Complementary DNA was synthesized using the PrimeScript™ RT reagent Kit (TaKaRa, Shiga, Japan). The expression of Hes1 was analyzed with the following primers: (forward primer 5′-AGCCCACCTCTCTCTTCTGA-3′, reverse primer 5′-AGGCGCAATCCAATATGAAC-3′) and GAPDH gene was used as an internal control. Real-time RT–PCR was performed on CFX96 Touch™ Real-Time PCR Detection System (Bio-Rad Laboratories, USA) using TB Green® Premix Ex Taq™ II (TaKaRa, Japan), and data were analyzed based on the formula of 2^−*ΔΔ*ct^.

### 2.7. ELISA for IL-17A

Serum- and cell-free supernatant samples were collected. IL-17A concentration was measured by ELISA kit (R&D Systems, Minneapolis, MN, USA) according to the manufacturer's instruction.

### 2.8. Western Analysis for IL-17A

Total protein from skin tissues was extracted and subjected to 10% sodium dodecyl sulphate-polyacrylamide gel electrophoresis. The resolved proteins were transferred to polyvinylidene difluoride membranes and blocked with 5% milk powder in Tris-buffered saline, then probed with anti-IL-17A primary antibody (at 1 : 1000 dilution, Affinity Biosciences, Cincinnati, OH, USA) and detected with a secondary antibody (Jackson ImmunoResearch Laboratories Inc., Pennsylvania, USA). Normalization was performed by blotting the same membranes with GAPDH antibody (Affinity Biosciences, USA). Image Lab™ software was used to obtain and quantify the IL-17A protein expression levels.

## 3. Statistical Analysis

According to the results of the normal distribution test (Shapiro–Wilk test), data were expressed as mean ± SD. The independent Samples *t*-test was used to analyze the difference of target lesion scores between the model group and intervention group. A one-way analysis of variance (ANOVA) followed by the LSD (least significant difference) test and Welch ANOVA followed by Tamhane's T2 test were used to compare the differences of other indexes among the three experimental groups. Statistical analysis was completed using SPSS and GraphPad Prism systems. A *P* value of <0.05 was considered statistically significant.

## 4. Results

### 4.1. Inhibiting Notch-Hes1 Signaling by DAPT Alleviated the Severity of Mouse Psoriasis-Like Skin Inflammation

**Figure 1:**
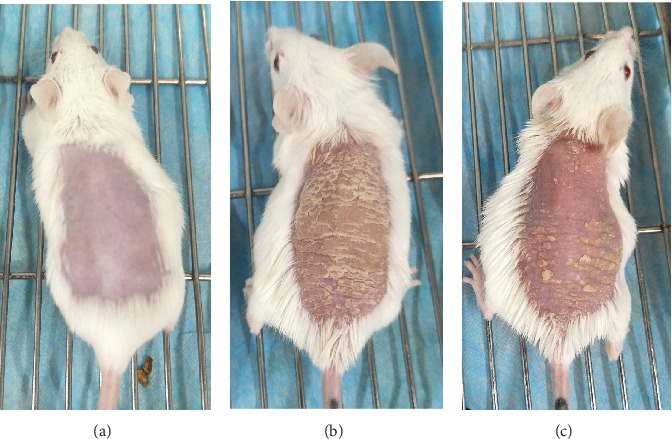
Changes of skin structural characters of experimental mice after consecutive 6 days' treatment. (a) Control mice did not show any sign of inflammation. (b) Model mice displayed significant signs of psoriasis-like inflammation. (c) Intervention mice presented similar change of psoriasis-like inflammation, while the degree of erythema, scaling, and thickening was obviously alleviated compared to model mice.

**Figure 2:**
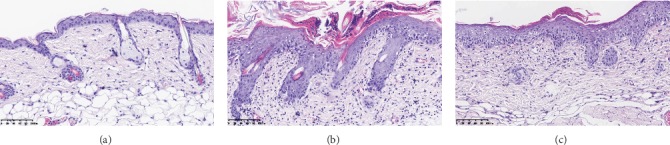
Histopathological changes of experimental mice after consecutive 6 days' treatment. (a) The epidermis of control mice was thin and consisted of only 1-2 layers of epidermal cells. (b) Model mice presented classic psoriasis-like histopathological features. (c) Intervention mice displayed significantly reduced epidermal hyperplasia and dermal inflammatory cell infiltration compared to model mice.

The control mice did not present any sign of skin inflammation during consecutive 6 days. Since the second day, model mice displayed the signs of psoriasis-like inflammation, such as erythema, scaling, and thickening on their shaved back skin, which got aggravated continually and achieved the most serious degree on the sixth day. Similar changes can also been found in intervention mice, but the severity was significantly alleviated compared to model mice (Figure 1). Correspondingly, the target lesion scores were significantly increased in model mice, while decreased in intervention mice (40.30 ± 2.75 vs. 28.30 ± 3.65, *t* = 8.298, *P* < 0.01). Histopathological examination of the mouse back skin showed that there were only 1-2 layers of epidermal cells in control mice. Model mice presented obviously epidermal hyperplasia, hyperkeratosis, parakeratosis with Munro microabscess, and trochanterellus extension, as well as dermal telangiectasias and massive inflammatory cell infiltration; all of which matched the characteristic histological picture of psoriasis. After DAPT treatment, the degree of epidermal hyperplasia and dermal inflammatory cell infiltration in intervention mice was significantly reduced (Figure 2). Furthermore, the thickness of epidermal cell layers was measured and compared, and the differences among the three experimental groups and between every two groups were all significant (Table 1).

### 4.2. Inhibiting Notch-Hes1 Signaling by DAPT Mitigated the Splenomegaly

**Figure 3:**
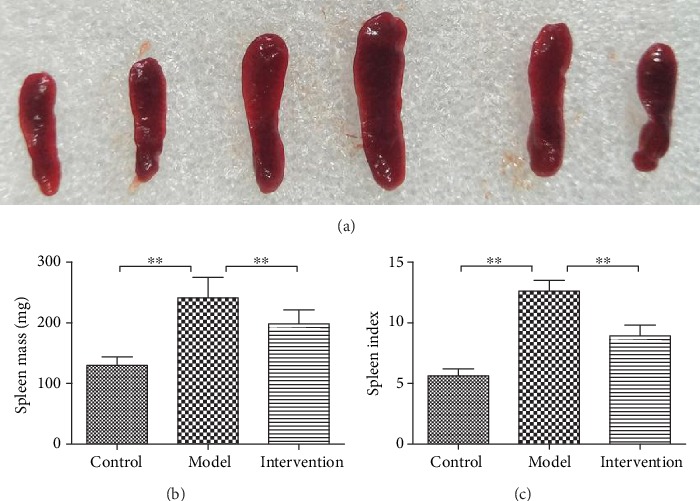
Spleen size, spleen mass, and spleen index of experimental mice after consecutive 6 days' treatment. (a) Comparison of spleen size among experimental mice. (b) Comparison of spleen mass among experimental mice (^∗∗^*P* < 0.01). (c) Comparison of spleen index among experimental mice (^∗∗^*P* < 0.01).

As shown in Figure 3, compared to control mice, the spleen size of model mice was significantly enlarged with an increased spleen mass and spleen index. While, after DAPT treatment, the spleen mass and spleen index of intervention mice were resultantly decreased.

### 4.3. Inhibiting Notch-Hes1 Signaling by DAPT Reduced the Elevated IL-17A^+^*γδ*^+^T Cell Percentage

**Figure 4:**
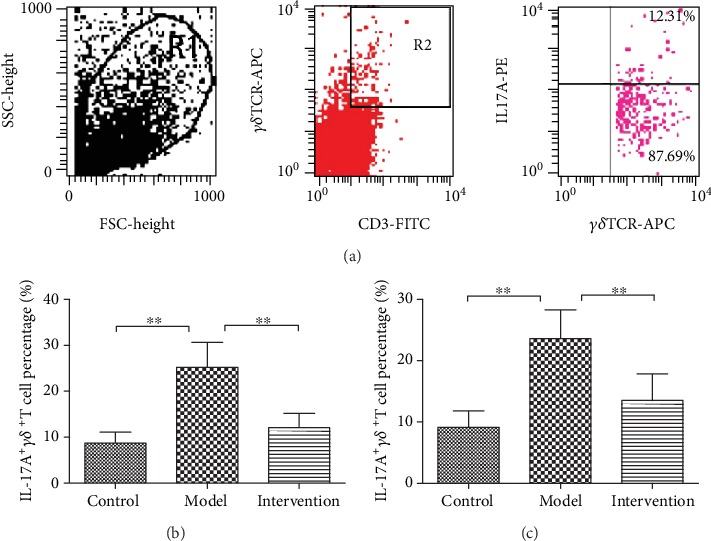
The proportion of splenic and skin IL-17A^+^*γδ*^+^T cells (IL-17A^+^*γδ*^+^T cells/*γδ*^+^T cells%). (a) Representative pictures for IL-17A^+^*γδ*^+^T cell percentage. (b) Comparison of splenic IL-17A^+^*γδ*^+^T cell percentage among experimental mice (^∗∗^*P* < 0.01). (c) Comparison of skin IL-17A^+^*γδ*^+^T cell percentage among experimental mice (^∗∗^*P* < 0.01).

Representative pictures for IL-17A^+^*γδ*^+^T cell percentage (IL-17A^+^*γδ*^+^T cells/*γδ*^+^T cells%) are presented in Figure 4(a). The proportions of IL-17A^+^*γδ*^+^T cells were significantly different among the three experimental mice both in spleen and skin tissues (spleen *F*′ = 37.49, *P* < 0.01, Figure 4(b); skin *F* = 34.04, *P* < 0.01, Figure 4(c)). Further comparison between every two groups showed that IL-17A^+^*γδ*^+^T cell percentage of model mice in spleen and skin samples was significantly higher than that of the control group (spleen 25.23 ± 5.42% vs. 8.73 ± 2.38%, *P* < 0.01; skin 23.58 ± 4.70% vs. 9.12 ± 2.70%, *P* < 0.01). While in DAPT-treated intervention mice, both the splenic and skin IL-17A^+^*γδ*^+^T cell percentages were markedly decreased compared to model mice (spleen 12.10 ± 3.09% vs. 25.23 ± 5.42%, *P* < 0.01; skin 13.51 ± 4.36% vs. 23.58 ± 4.70%, *P* < 0.01).

### 4.4. Inhibiting Notch-Hes1 Signaling by DAPT Downregulated the Increased Hes1 mRNA Expression

**Figure 5:**
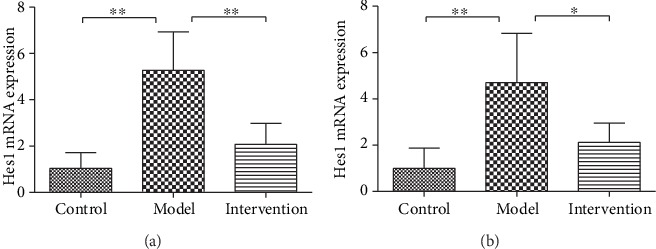
The Hes1 mRNA expression of splenic and skin tissues. (a) Comparison of splenic Hes1 mRNA expression among experimental mice (^∗∗^*P* < 0.01). (b) Comparison of Hes1 mRNA expression in skin tissues among experimental mice (^∗∗^*P* < 0.01, ^∗^*P* < 0.05).

There were significant differences of splenic and skin Hes1 mRNA expression among the three experimental groups (spleen *F*′ = 27.10, *P* < 0.01, Figure 5(a); skin *F*′ = 13.76, *P* < 0.01, Figure 5(b)), and further comparison displayed that Hes1 mRNA expression was obviously upregulated in model mice compared to control mice (spleen 5.27 ± 1.67 vs. 1.03 ± 0.69, *P* < 0.01; skin 4.71 ± 2.12 vs. 1.00 ± 0.88, *P* < 0.01). After DAPT treatment, Hes1 mRNA expression of intervention mice was dramatically decreased (spleen 2.07 ± 0.92 vs. 5.27 ± 1.67, *P* < 0.01; skin 2.12 ± 0.83 vs. 4.71 ± 2.12, *P* < 0.05).

### 4.5. Inhibiting Notch-Hes1 Signaling by DAPT Decreased IL-17A Expression and Secretion

**Figure 6:**
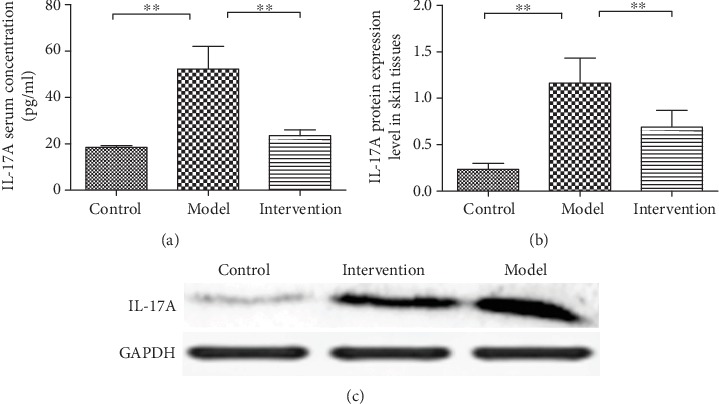
IL-17A serum concentration and protein expression level in skin tissues. (a) Comparison of serum IL-17A concentration among experimental mice (^∗∗^*P* < 0.01). (b) Comparison of IL-17A protein expression level in skin tissues among experimental mice (the expression of IL-17A was calculated after normalization to GADPH expression (^∗∗^*P* < 0.01). (c) Representative picture for IL-17A protein expression level.

There were significant differences of IL-17A serum concentration among the three experimental groups (*F*′ = 71.75, *P* < 0.01, Figure 6(a)). IL-17A serum concentration of model mice was significantly higher than that of control mice (52.20 ± 9.79 pg/ml vs. 18.42 ± 0.78 pg/ml, *P* < 0.01). Consistently, the protein expression level of IL-17A in skin tissues was significantly different among the three experimental groups (F′ = 74.78, *P* < 0.01, Figure 6(b)), and the expression level of the model group was markedly increased compared to control (1.16 ± 0.27 vs. 0.23 ± 0.07, *P* < 0.01). However, DAPT treatment can obviously suppress IL-17A expression and secretion, which resulted to a significantly decreased IL-17A concentration and protein expression in serum and skin sample, respectively (IL-17A serum concentration, 23.48 ± 2.57 pg/ml vs. 52.20 ± 9.79 pg/ml; IL-17A protein expression level, 0.69 ± 0.18 vs. 1.16 ± 0.27, both *P* < 0.01).

### 4.6. Inhibiting Notch-Hes1 Signaling by DAPT Decreased IL-17A^+^*γδ*^+^T Cell Percentage and IL-17A Secretion in a Dose-Dependent Manner

**Figure 7:**
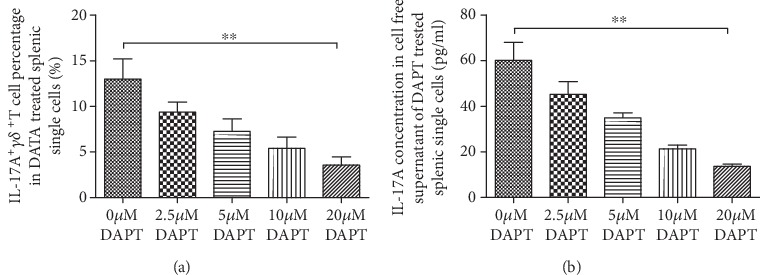
IL-17A^+^*γδ*^+^T cell percentage in DAPT-treated splenic single cells and IL-17A concentration in cell-free supernatant Both IL-17A^+^*γδ*^+^T cell percentage (a) and IL-17A concentration (b) presented a dose-dependent decrease with the increased concentration of DAPT in cultured splenic single cells.

The in vitro study showed that IL-17A^+^*γδ*^+^T cell percentage and IL-17A secretion in DAPT-treated splenic single cells and cell-free supernatant presented a dramatically reduced trend with the increased concentration of DAPT (IL-17A^+^*γδ*^+^T cell percentage *F*′ = 33.05, *P* < 0.01, Figure 7(a); IL-17A concentration *F* = 61.08, *P* < 0.01, Figure 7(b)].

## 5. Discussion

Growing evidence indicated that psoriasis is strongly associated with IL-17A, and clinically, the monoclonal antibodies to IL-17A or its receptor IL-17R showed a dramatic effect against psoriasis [2–4, 6–12]. Recently, *γδ*T cells have been reported to be a major IL-17A producer in human psoriatic lesion and mouse psoriasis-like skin inflammation [18]. In the present study, the increased IL-17A^+^*γδ*^+^T cell percentage was also found in spleen and skin lesions of psoriatic mice model, which further confirmed the important role of IL-17A^+^*γδ*^+^T cells in the pathogenesis of psoriasis.

Notch signaling plays a pivotal role in cell fate decision and lineage commitment of lymphocytes; more importantly, it is also well-known for its role in the development of *αβ* T cells and its requirement for *γδ*T cell development [29–31]. The interaction between Notch ligands and Notch receptors can initiate a proteolytic cleavage of the transmembrane Notch peptide near the extracellular surface, which, in turn, induces a conformational change to allow cleavage of the Notch transmembrane domain by the *γ*-secretase, then results in the release of an intracellular Notch fragment (NICD). NICD rapidly translocates to the nucleus and interacts with the DNA binding protein known as CSL (CBF-1, suppressor of hairless, Lag-1), then exerts a biological effect by activating a target gene (such as Hes1). Therefore, the activation of Notch signaling is dependent on *γ*-secretase, a multimeric membrane protein complex composed of presenilin, nicastrin, Aph-1, and Pen-2, among which presenilin represents the catalytic center of *γ*-secretase. DAPT is one of the major *γ*-secretase inhibitors of dipeptidic type and commonly used to block Notch signaling, which can target the C-terminal fragment of presenilin, then effectively block presenilin, inhibit the release of active NICD, and result in the downstream signal molecules downregulated or static [32]. Notch signaling has been shown to function in the development, differentiation, and activation of T cells [33–35]. The effect of *γ*-secretase inhibitors on human and murine production of IL-17A has been tested by treating Th17-polarized cells with another two kinds of *γ*-secretase inhibitors, IL-CHO, and compound E, which results in significantly reduced IL-17A levels in comparison with DMSO-treated Th17-polarized cells [36]. Our previous studies have also demonstrated that DAPT treatment can significantly decreased IL-17A expression and secretion of CD4^+^ T cells cultured in Th17 polarizing conditions both in psoriatic patients and model mice [27, 37]. In the present study, we found increased IL-17A^+^*γδ*^+^T cell proportion, IL-17A serum concentration, and IL-17A protein expression level of skin lesion in psoriatic model mice and blocking Notch signaling by DAPT resulted in an obvious decrease of the above indexes as well as strong alleviation of psoriatic-like lesion, thickened epidermal cell layers, and dermal inflammatory cell infiltration in mice of the intervention group. To further evaluate the inhibitory effect of blocking Notch-Hes1 signaling on IL-17A^+^*γδ*^+^T cell differentiation and function, splenic single cells of model mice were treated by different concentrations of DAPT; in accordance with the results of in vivo intervention study, blockade of Notch-Hes1 signaling can inhibit the expression of IL-17A^+^*γδ*^+^T cells and the secretion of IL-17A in a dose-dependent matter. So combination of in vitro and in vivo results, we may conclude that Notch-Hes1 signaling can express its effects in the pathogenesis of psoriasis by regulating IL-17A^+^*γδ*^+^T cells.

Notch1 binding to putative CSL binding sites in human IL-17 promoter has been demonstrated [36]. Though Th17 cells and IL-17A^+^*γδ*^+^T cells are two major IL-17A producers in psoriasis, their differentiation mechanism is different. In Th17 cells, signal transducer and activator of transcription-3 (STAT3) play an important role in transducing IL-6 receptor signaling, while IL-6 is not required for IL-17A^+^*γδ*^+^T cells and STAT3 is dispensable for the development of IL-17A^+^*γδ*^+^T cells, which can develop in STAT3-deficient mice [28, 38]. ROR*γ*t, a key transcription factor, is indispensable for the differentiation of Th17 cells. Both mouse CD4^+^ T cells treated with *γ*-secretase inhibitors and human CD4^+^ T cells nucleoporated with Notch1-specific siRNA under Th17 polarizing conditions presented significantly decreased levels of ROR*γ*t transcripts [36]. In addition, Notch1 can bind directly to putative CSL binding sites in human ROR*γ*t promoter, and the binding site CSL1 can be inhibited by treatment with *γ*-secretase inhibitors [36], while the expression level of ROR*γ*t is not necessarily related to IL-17 production in *γδ*T cells, and ROR*γ*t is only partly required for intrathymic development of IL-17A^+^*γδ*^+^T cells [28, 39]. Hes1 is an important target molecule of Notch signaling; more importantly, it is critically involved in the development of IL-17A^+^*γδ*^+^T cells [28]. The absolute number of *γδ*T cells in the thymus of Hes1-deficient mice was lower, and IL-17-producing *γδ*T cells were even more strikingly decreased [28]. In the periphery, IL-17 production by splenic *γδ*T cells was significantly reduced in the absence of Hes1, while the number of IL-17-producing *γδ*TCR^−^ splenocytes, most of which were CD4^+^*αβ* T cells, was unaffected by the absence of Hes1 [28]. In our present study, we found that the Hes1 mRNA expression level were increased both in spleen and skin lesions of psoriatic mice model, and in parallel with IL-17A^+^*γδ*^+^T cell proportion, blockade of Notch signaling can significantly decreased the mRNA expression level of Hes1, which is consistent with the former report that Hes1 expression was correlated with IL-17 production of *γδ*T cells [28]. Therefore, taking into the literature reports and our research results into account, we may conclude that Notch-Hes1 signaling can regulate the two major sources of IL-17A, Th17 cells, and IL-17A^+^*γδ*^+^T cells, in psoriasis through different transcription factor pathway, respectively.

Hyperproliferation and aberrant differentiation are important characters of psoriatic lesions, which may be maintained by some cytokines, including IL-17A [40]. Within psoriatic lesions, keratinocytes are the main cell type that expresses IL-17R [15]. Alone and in combination with other cytokines, such as IFN-*γ*, IL-22, and TNF, IL-17A can cause keratinocyte proliferation as well as the production of chemokine, cytokine, and antimicrobial peptides, which becomes a self-amplifying loop and acts back on the dendritic cells, T cells, and neutrophils to perpetuate the cutaneous inflammatory process [40, 41]. Upexpressed Notch receptors and target gene Hes1 have been found in psoriatic epidermis, and the activation of Notch signaling is involved in keratinocyte differentiation [20, 42]. Moreover, Notch signaling activation in the epidermal basal layer may induce a remarkable dermal T cell infiltration, which indicates a Notch-dependent interaction between epidermal and dermal compartments [43]. In this present study, we also displayed a significantly thickened epidermal cell layer and dermal inflammatory cell infiltration in model mice, while blocking Notch-Hes1 signaling by DAPT, the thinning of psoriatic lesions was demonstrated not only in general observation but also in histopathological change of epidermal cell layers; moreover, dermal inflammatory cell infiltration was also obviously alleviated. Then, to a certain extent, we may conclude that the abnormal keratinocyte differentiation and inflammatory cell infiltration of psoriatic lesions may be regulated directly and indirectly by the regulating axis of Notch-Hes1 signaling through IL-17A.

## 6. Conclusions

In summary, the current study may suggest that Notch-Hes1 signaling is participated in the pathogenesis of psoriasis by regulating IL-17A^+^*γδ*^+^T cell expression and IL-17A secretion, and blocking Notch-Hes1 signaling may be a new strategy for immunotherapy of psoriasis.

## Figures and Tables

**Table 1:** Comparison of epidermal cell layers among experimental mice.

	Control mice	Model mice	Intervention mice
Epidermal cell layers thickness (*μ*m)	14.30 ± 1.70	86.41 ± 12.67	42.65 ± 7.10
	*F*′ = 112.45, *P* < 0.01		

## Data Availability

The data used to support the findings of this study are available from the corresponding author upon request.

## References

[1] Schön M. P., Boehncke W. H. (2005). Psoriasis. *New England Journal of Medicine*.

[2] Malakouti M., Brown G. E., Wang E., Koo J., Levin E. C. (2015). The role of IL-17 in psoriasis. *Journal of Dermatological Treatment*.

[3] Hawkes J. E., Chan T. C., Krueger J. G. (2017). Psoriasis pathogenesis and the development of novel targeted immune therapies. *Journal of Allergy and Clinical Immunology*.

[4] Lynde C. W., Poulin Y., Vender R., Bourcier M., Khalil S. (2014). Interleukin 17A: toward a new understanding of psoriasis pathogenesis. *Journal of the American Academy of Dermatology*.

[5] van der Fits L., Mourits S., Voerman J. S. A. (2009). Imiquimod-induced psoriasis-Like Skin Inflammation in Mice is Mediated via the IL-23/IL-17 axis. *Journal of Immunology*.

[6] Reis J., Vender R., Torres T. (2019). Bimekizumab: The First Dual Inhibitor of Interleukin (IL)-17A and IL-17F for the Treatment of Psoriatic Disease and Ankylosing Spondylitis. *BioDrugs*.

[7] Frieder J., Kivelevitch D., Menter A. (2017). Secukinumab: a review of the anti-IL-17A biologic for the treatment of psoriasis. *Therapeutic Advances in Chronic Disease*.

[8] Gordon K. B., Blauvelt A., Papp K. A. (2016). Phase 3 Trials of Ixekizumab in Moderate-to-Severe Plaque Psoriasis. *New England Journal of Medicine*.

[9] Griffiths C. E., Reich K., Lebwohl M. (2015). Comparison of ixekizumab with etanercept or placebo in moderate-to-severe psoriasis (UNCOVER-2 and UNCOVER-3): results from two phase 3 randomised trials. *Lancet*.

[10] Reich K., Pinter A., Lacour J. P. (2017). Comparison of ixekizumab with ustekinumab in moderate-to-severe psoriasis: 24-week results from IXORA-S, a phase III study. *British Journal of Dermatology*.

[11] Paul C., Griffiths C. E. M., van de Kerkhof P. C. M. (2019). Ixekizumab provides superior efficacy compared with ustekinumab over 52 weeks of treatment: results from IXORA-S, a phase 3 study. *Journal of the American Academy of Dermatology*.

[12] Bilal J., Berlinberg A., Bhattacharjee S., Trost J., Riaz I. B., Kurtzman D. J. B. (2018). A systematic review and meta-analysis of the efficacy and safety of the interleukin (IL)-12/23 and IL-17 inhibitors ustekinumab, secukinumab, ixekizumab, brodalumab, guselkumab and tildrakizumab for the treatment of moderate to severe plaque psoriasis. *Journal of Dermatological Treatment*.

[13] Di Cesare A., Di Meglio P., Nestle F. O. (2009). The IL-23/Th17 axis in the immunopathogenesis of psoriasis. *Journal of Investigative Dermatology*.

[14] Lowes M. A., Kikuchi T., Fuentes-Duculan J. (2008). Psoriasis vulgaris lesions contain discrete populations of Th1 and Th17 T cells. *Journal of Investigative Dermatology*.

[15] Kim J., Krueger J. G. (2015). The immunopathogenesis of psoriasis. *Dermatologic Clinics*.

[16] Korn T., Bettelli E., Oukka M., Kuchroo V. K. (2009). IL-17 and Th17 Cells. *Annual Review of Immunology*.

[17] Res P. C. M., Piskin G., de Boer O. J. (2010). Overrepresentation of IL-17A and IL-22 Producing CD8 T Cells in Lesional Skin Suggests Their Involvement in the Pathogenesis of Psoriasis. *PLoS One*.

[18] Cai Y., Shen X., Ding C. (2011). Pivotal Role of Dermal IL-17-Producing *γδ* T Cells in Skin Inflammation. *Immunity*.

[19] Hansson E. M., Lendahl U., Chapman G. (2004). Notch signaling in development and disease. *Seminars in Cancer Biology*.

[20] Nowell C., Radtke F. (2013). Cutaneous Notch Signaling in Health and Disease. *Cold Spring Harbor Perspectives in Medicine*.

[21] Abdou A. G., Maraee A. H., Sharaf A., Elnaidany N. F. (2012). Up-regulation of Notch-1 in psoriasis: an immunohistochemical study. *Annals of Diagnostic Pathology*.

[22] Lowell S., Jones P., Le Roux I., Dunne J., Watt F. M. (2000). Stimulation of human epidermal differentiation by Delta-Notch signalling at the boundaries of stem-cell clusters. *Current Biology*.

[23] Rooney P., Connolly M., Gao W. (2014). Notch-1 mediates endothelial cell activation and invasion in psoriasis. *Experimental Dermatology*.

[24] Gao W., Sweeney C., Walsh C. (2013). Notch signalling pathways mediate synovial angiogenesis in response to vascular endothelial growth factor and angiopoietin 2. *Annals of the Rheumatic Diseases*.

[25] Auderset F., Coutaz M., Tacchini-Cottier F. (2012). The role of Notch in the differentiation of CD4^+^ T helper cells. *Current Topics in Microbiology and Immunology*.

[26] Bailis W., Yashiro-Ohtani Y., Fang T. C. (2013). Notch Simultaneously Orchestrates Multiple Helper T Cell Programs Independently of Cytokine Signals. *Immunity*.

[27] Ma L., Xue H., Gao T., Gao M., Zhang Y. (2018). Notch1 Signaling Regulates the Th17/Treg Immune Imbalance in Patients with Psoriasis Vulgaris. *Mediators of Inflammation*.

[28] Shibata K., Yamada H., Sato T. (2011). Notch-Hes1 pathway is required for the development of IL-17-producing *γδ* T cells. *Blood*.

[29] Gogoi D., Dar A. A., Chiplunkar S. V. (2014). Involvement of Notch in Activation and Effector Functions of *γδ* T cells. *Journal of Immunology*.

[30] Hozumi K., Mailhos C., Negishi N. (2008). Delta-like 4 is indispensable in thymic environment specific for T cell development. *Journal of Experimental Medicine*.

[31] Radtke F., Wilson A., Stark G. (1999). Deficient T Cell Fate Specification in Mice with an Induced Inactivation of Notch1. *Immunity*.

[32] Morohashi Y., Kan T., Tominari Y. (2006). C-terminal Fragment of Presenilin Is the Molecular Target of a Dipeptidic *γ*-Secretase-specific Inhibitor DAPT (N-[N-(3,5-Difluorophenacetyl)-L-alanyl]-S-phenylglycinet-Butyl Ester). *Journal of Biological Chemistry*.

[33] Fiúza U. M., Arias A. M. (2007). Cell and molecular biology of notch. *Journal of Endocrinology*.

[34] Okamoto M., Matsuda H., Joetham A. (2009). Jagged1 on dendritic cells and Notch on CD4+ T cells initiate lung allergic responsiveness by inducing IL-4 production. *Journal of Immunology*.

[35] Eagar T. N., Tang Q., Wolfe M., He Y., Pear W. S., Bluestone J. A. (2004). Notch 1 Signaling Regulates Peripheral T Cell Activation. *Immunity*.

[36] Keerthivasan S., Suleiman R., Lawlor R. (2011). Notch signaling regulates mouse and human Th17 differentiation. *Journal of Immunology*.

[37] Ma L., Xue H., Qi R., Wang Y., Yuan L. (2018). Effect of *γ*-secretase inhibitor on Th17 cell differentiation and function of mouse psoriasis-like skin inflammation. *Journal of Translational Medicine*.

[38] Lochner M., Peduto L., Cherrier M. (2008). In vivo equilibrium of proinflammatory IL-17+ and regulatory IL-10+ Foxp3+ RORgamma t+ T cells. *Journal of Experimental Medicine*.

[39] Ivanov I. I., McKenzie B. S., Zhou L. (2006). The Orphan Nuclear Receptor ROR*γ*t Directs the Differentiation Program of Proinflammatory IL-17+ T Helper Cells. *Cell*.

[40] Lowes M. A., Suárez-Fariñas M., Krueger J. G. (2014). Immunology of psoriasis. *Annual Review of Immunology*.

[41] Chiricozzi A., Guttman-Yassky E., Suárez-Fariñas M. (2011). Integrative responses to IL-17 and TNF-*α* in human keratinocytes account for key inflammatory pathogenic circuits in psoriasis. *Journal of Investigative Dermatology*.

[42] Rangarajan A., Talora C., Okuyama R. (2001). Notch signaling is a direct determinant of keratinocyte growth arrest and entry into differentiation. *EMBO Journal*.

[43] Ambler C. A., Watt F. M. (2010). Adult epidermal Notch activity induces dermal accumulation of T cells and neural crest derivatives through upregulation of jagged 1. *Development*.

